# The Need of a Medical Reference Library

**Published:** 1888-11

**Authors:** 


### THE NEED OF A MEDICAL REFERENCE LIBRARY.

At the present time there is considerable attention concentrated upon the Newberry Library, which is being organized here, and an effort is being made to secure the establishment of a medical department in it. It is understood that this is to be strictly a reference library. The endowment is ample to make it complete in all departments of science and art, if the money be properly expended.

There are two things that are essential to any important library—books and a capable librarian. For the Newberry Library such a librarian has been secured, and if too much money be not expended in monumental buildings, as has too often happened, so that the essentials have ever afterward suffered, the Newberry Library may be made of inestimable value for reference. It is very desirable that such a mistake be not repeated here, as making the building of first importance and its contents a secondary consideration.

Chicago has become an important medical centre, with several medical colleges, and the medical men resident here need the advantages which a large medical library of carefully selected books of reference would afford. Physicians from other points, especially in the interior of our country, would also find it of great assistance to them in their literary work. The library of the Surgeon-General's office of the army in Washington affords such facilities, especially for Eastern physicians, but it is almost inaccessible, because of its remoteness, for physicians residing in the interior. The present occasion affords an opportunity of supplying this need, and the trustees of the Newberry Library should recognize the fact and provide for a medical department, to be in keeping with what it is proposed to make the other departments. It would be incomplete without a medical department thus equipped. Access to such a medical library would not only be a great convenience, but the advantage to medical science would be incalculable.

The State has appropriated money for moderate libraries in some of its charitable institutions, but political and other considerations led to the locating of these institutions in various parts of the State, and the libraries are not available except to a very limited number. Chicago has never made a successful effort to build up a medical library, and there is now no collection of medical books in the city sufficiently complete for reference. In the Chicago Public Library there is a limited medical department, containing books obtained from various sources, but it is without a special librarian devoted to its care and prosperity alone. With the co-operation of the profession, and the library officials, this incomplete collection of books could be made a servicable library in time, but probably not such a reference library as is needed. A librarian devoted especially to the interests of the medical department is a requisite. The profession should support the administration in its efforts to increase the usefulness of this department, and aid in contributing to it, even more than it has already done. The library should contain the current American and foreign serials in medicine and biology in different languages. These journals should always be accessible.

Complete files of the same serials, running back as far as possible, would be a desirable acquisition, and a librarian devoted to this department alone could do much to secure them and other matter of various kinds that would materially increase the value of the present medical department for the use of the medical profession.

